# Chronic Q Fever Presenting as a Contained Rupture of an Infected Native Aortic Aneurysm: A Case Report

**DOI:** 10.1016/j.ejvsvf.2025.11.003

**Published:** 2025-11-20

**Authors:** Lea Schacher, Laina Passos, Michel Bosiers, Vladimir Makaloski, Daniel Becker, Drosos Kotelis

**Affiliations:** a Department of Vascular Surgery, Swiss Aortic Centre Bern, Bern University Hospital, University of Bern, Bern, Switzerland; b Faculty of Medicine, University of Basel, Basel, Switzerland

**Keywords:** Chronic Q fever, *Coxiella burnetti* (*C. burnetti*), Infected native aortic aneurysm (INAA), Q fever, Ruptured aortic aneurysm

## Abstract

**Introduction:**

*Coxiella burnetti* (*C. burnetii*) is the causative agent of Q fever. Chronic Q fever may lead to various complications, including infected native aortic aneurysms (INAA). Both INAA and chronic Q fever typically present with non-specific symptoms, often resulting in delayed diagnosis. While current guidelines provide recommendations for the management of INAA, there are no specific or standardised protocols addressing INAA in the context of chronic *C. burnetii* infection. However, antimicrobial therapy should be directed towards the causative pathogen. This case report describes a rare case of INAA secondary to chronic Q fever.

**Case:**

A 64 year old man presented with lumbar pain due to contained rupture of a juxtarenal abdominal aortic aneurysm. Urgent open surgical repair with inter-renal clamping, resection of infected tissue, reconstruction with pericardial tube graft and omental flap plasty was performed due to suspicion of infection. Intra-operative samples revealed *C. burnetii* to be the causative agent of chronic Q fever. Oral antibiotic treatment with doxycycline 100 mg twice daily and hydroxychloroquine 200 mg thrice daily was established.

**Discussion:**

*C. burnetii* infection should be considered as a possible pathogen in a case of culture negative INAA. Early diagnosis, open surgical treatment using bovine material and omental flap plasty, prolonged targeted antimicrobial therapy, and follow up assessment with blood samples and computed tomography scans in close cooperation with infectious disease specialists are recommended to improve outcomes in these rare and challenging cases. Case reports such as this contribute to raising clinical awareness and provide valuable insights into the absence of standardised treatment guidelines.

## HIGHLIGHTS

• We report a rare case of chronic subfebrile infection due to Coxiella burnetti, complicated by an infective native aortic aneurysm with a chronic contained rupture.
• The case illustrates the diagnostic challenges of chronic Q fever associated vascular infection and highlights the role of pathogen identification in guiding management.
• Successful treatment was achieved through combined surgical repair and prolonged targeted antibiotic ther

## INTRODUCTION

Infected native aortic aneurysms (INAA) represent a specific subset of abdominal aortic aneurysms. Patients with INAA are typically symptomatic, most commonly presenting with pain and fever. Less specific systemic symptoms, such as fatigue and malaise, may also occur. Laboratory findings frequently reveal elevated inflammatory markers, including C reactive protein (CRP) and leucocytosis. Contrast enhanced computed tomography (CT) is the imaging modality of choice for diagnosis. While blood cultures may assist in identifying the causative organism, they do not need to be positive to establish the diagnosis of an INAA; however, their collection remains mandatory.^1^

*Coxiella burnetti* (*C. burnetii*), the causative agent of Q fever, is a rare but notifiable pathogen in Switzerland, with a national incidence of 1.63 per 100 000 people in 2024.^2^ Acute Q fever commonly presents with non-specific symptoms such as isolated fever, atypical pneumonia, and hepatitis. Chronic Q fever, on the other hand, is known to cause seronegative endocarditis and has also been implicated in various vascular complications, including INAA.^3^ *C. burnetii*, being an intracellular pathogen, is often culture negative and may therefore be overlooked as a causative agent of culture negative INAA. These aneurysms may be located in either the abdominal or thoracic aorta and are often associated with underlying atherosclerotic disease or previous vascular grafts.^4^^,^^5^ However, cases have been reported where no identifiable predisposing vascular condition was present.^5^

This case report describes a rare case of a patient presenting with lumbar pain and being found to have a chronically contained rupture of an INAA secondary to chronic *C. burnetii* (Q fever) infection. Written informed consent was obtained from the patient for the publication of this case report.

## REPORT

**Figure 1:**
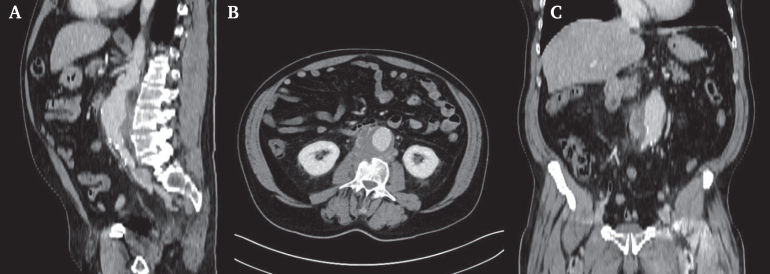
Pre-operative, contrast enhanced computed tomography showing in the (A) sagittal, (B) axial and (C) coronal planes, a juxtarenal aortic aneurysm with an oedematous fluid collection posteriorly.

**Figure 2:**
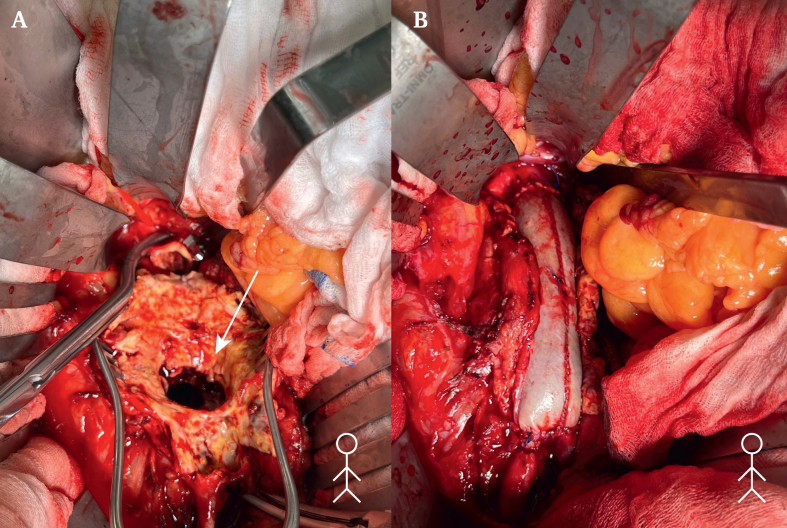
Intra-operative documentation of (A) the contained rupture in the posterior aortic wall and (B) the bovine tube graft after implantation, before coverage with omentum plasty.

A 64 year old man was referred to the emergency department, from a primary care hospital, with suspected INAA. He presented as haemodynamically stable, afebrile (blood pressure 158/84 mmHg, heart rate 97 bpm), complaining of worsening chronic back pain over the previous four months. His medical history included a 50 pack year smoking history, chronic obstructive pulmonary disease, arterial hypertension, and prior degenerative disease with foraminal compression at L4 on magnetic resonance imaging six years earlier. Further history revealed night sweats and shivering over the previous three months, a cat bite in the same timeframe, and travel to Central America two years before. Initial CT imaging showed a juxtarenal abdominal aortic aneurysm (JAAA) with a contained posterior rupture and associated fluid collections involving the L3 vertebra and right psoas muscle suspicious of being associated with infection (Fig. 1). Due to suspicion of infection per Delphi criteria^1^ and pending culture results, open surgical repair was performed via midline laparotomy. After opening the retroperitoneum, both renal arteries and proximal common iliac arteries were exposed for clamping. Systemic heparin was administered, and inter-renal clamping (above left and below right renal artery) was performed. After longitudinal incision of the aneurysm and removal of the haematoma, a large chronic contained rupture extending toward the vertebral column was revealed; aortic wall and thrombotic material was sent for microbiological analysis, which later yielded negative results. A bovine pericardial tube graft, tailored to approximately three times the diameter of the native healthy aorta, was anastomosed to the infrarenal aorta in an inlay technique using a single running suture line with 4–0 Prolene (Fig. 2). The posterior aortic wall was additionally reinforced with a bovine pericardial strip. Distally, the graft was anastomosed to the bifurcation of the aorta, using running 4–0 Prolene sutures.

The inferior mesenteric artery, which back bled profusely, was ligated. Extensive irrigation with Microdacin (Oculus Innovative Sciences, Petaluma, CA, USA) was performed, followed by omental coverage of the bovine reconstruction. The inter-renal aortic clamping phase lasted 52 minutes and estimated blood loss was 1.300 mL. The aortic wall was noted to be markedly fragile, consistent with infection related tissue degeneration.

Post-operatively, the patient developed fever (peak 39.2 °C). After consultation with infectious disease specialists, broad spectrum antibiotics (co-amoxicillin and vancomycin) were initiated after blood cultures, which turned out to be negative, were obtained. This finding reflects the well documented culture negative nature of *C. burnetii* infection and underscores the importance of considering this pathogen in culture negative INAA, where diagnosis relies on serology and, when available, CRP rather than conventional blood cultures. Despite persistent sub-febrile temperatures, his condition remained stable. On post-operative day 10, serology confirmed chronic *C. burnetii* (Q fever) infection with elevated phase 1 and 2 IgG titres (1:4096). Following consultation of the infectious disease specialists, oral antibiotic therapy was transitioned to doxycycline 100 mg bd and hydroxychloroquine 200 mg tds with a planned treatment duration of 24 months and regular serological monitoring. PCR of intra-operative thrombotic material later confirmed *C. burnetii*. The patient recovered without complications and was discharged to rehabilitation on post-operative day 13.

After discharge, the patient was monitored jointly by vascular surgery and infectious disease specialists. Long term oral therapy with doxycycline and hydroxychloroquine was continued, with dose adjustment, under regular therapeutic drug monitoring and *Coxiella* serology every three months in the first year. At six weeks he was stable without signs of recurrent infection, with a CRP of 16 mg/L and normal leukocyte count. At four months, follow up CT demonstrated stable peri-prosthetic inflammatory changes adjacent to the graft and stable osteolysis at L4, without new peri-prosthetic collections. No anastomotic insufficiency was observed. The patient remains alive and clinically stable five months after surgery.

## DISCUSSION

Chronic *C. burnetii* infection (Q fever) is a rare but important INAA aetiological agent. Transmission of *C. burnetii* is typically airborne, originating from domestic or wild animals, particularly during parturition. Known sources include contaminated dust, faeces, urine, and milk products from infected animals.^3^ Although traditionally associated with rural environments, the durability of *C. burnetii* in the environment and its ability to be aerosolised over large distances means that infection can occur even in the absence of direct animal contact.^5^ It is believed that there are currently no comprehensive data on the geographical distribution or transmission pathways of *C. burnetii* in Switzerland. The largest outbreak in Europe to date occurred in the Netherlands between 2007 and 2010.^5^

The clinical presentation of chronic Q fever is often non-specific. In this case, the patient had a history of a recent cat bite, previous travel to Central America, and several months of constitutional symptoms, including night sweats and chills, all of which are non-specific. Symptoms such as low grade fever, abdominal or lumbar pain, fatigue, and night sweats are common but not pathognomonic, potentially contributing to delayed or missed diagnoses.^4^ As a result, in order to optimise diagnosis, routine screening for chronic Q fever has been proposed in patients with unexplained culture negative aortic aneurysms and suspicion of being an INAA or vascular infections, particularly in endemic areas.^6^

The exact mechanism by which *C. burnetii* invades vascular structures remains poorly understood. A contiguous spread from the aortic wall to adjacent tissues is considered likely in some cases.^7^ In this patient, the aortic wall was the sole infected structure, although imaging showed inflammatory changes in the psoas muscle and L3 vertebra, possibly indicating early contiguous extension, which is in line with previous reports describing chronic Q fever as a cause of aortic aneurysms and acute ruptures,^5^ as well as other complications such as osteomyelitis and psoas abscesses.^7^ Like other primary aortic infections, it has been proposed that underlying vascular disease serves as a target for the *C. burnetii* infection.^4^^,^^5^ In this patient, CT findings revealed such arteriosclerotic changes, suggestive of an underlying vascular disease as a predisposing factor.

The diagnosis of INAA consists of patient history, clinical findings, laboratory and culture results, and may be suspected on CT when characteristic findings are present. These include peri-aortic inflammatory changes, irregular or contrast enhancing aneurysm walls, disruption of wall calcification, peri-aortic lymphadenopathy, and saccular or lobular aneurysmal morphology with lumen dilation.^8^ Once INAA is suspected, current guidelines recommend prompt initiation of empiric broad spectrum antibiotic therapy in accordance with local and patient factors, targeting common pathogens such as *Staphylococcus aureus* and Gram negative bacilli, alongside urgent surgical intervention (Class 1C recommendation).^9^ Of the 44 published cases of vascular complications associated with chronic Q fever,^4^^,^^7^ all patients received antibiotic therapy with doxycycline 200 mg/day, either as monotherapy or, in 28 cases, in combination with hydroxychloroquine 600 mg/day. This regimen was administered either for the entire treatment course or initiated after a switch from doxycycline monotherapy. In line with these reports, this patient was also treated with oral doxycycline in combination with oral hydroxychloroquine, with a planned treatment duration of 18–24 months.

According to current evidence and guideline recommendations, both open surgical and endovascular repair represent viable approaches for the management of aortic infections associated with aneurysm or dissection.^10^ *In situ* reconstruction is generally favoured over extra-anatomic repair in both aneurysms and ruptures, as it offers greater versatility and lower complication rates, although implantation of foreign material in an infected field remains a concern.^10^ In this case of chronic rupture of an infected native aortic aneurysm, open *in situ* repair was considered the most appropriate approach.

Of the referenced cases, 29 underwent surgical treatment. The mortality rate has previously been reported at approximately 25% at a follow up of at least three years and with diverse treatment measures;^4^ no new data contradicting these figures were found. Notably, this patient demonstrated a favourable outcome, with satisfactory recovery during follow up and no evidence of recurrent infection or vascular complications to date.

### Conclusion

This case of a ruptured JAAA due to chronic *C. burnetii* infection is a rare but clinically relevant aetiology of INAA. Non-specific symptoms such as fever and reduced clinical conditions with characteristic radiological findings should raise suspicion of INAA, leading to collection of blood cultures including screening for *C. burnetti* antibodies on PCR. Treatment should be based on an interdisciplinary approach between vascular surgeons and infectious disease specialists. Early diagnosis, open surgical repair with bovine material and omental flap plasty, and prolonged pathogen directed antimicrobial therapy appear essential to achieve favourable outcomes. Chronic Q fever should be considered in suspected INAAs that are culture negative.

## CONFLICT OF INTEREST

The authors have no conflicts of interest to declare.
